# Brief pain re-assessment provided more accurate prognosis than baseline information for low-back or shoulder pain

**DOI:** 10.1186/s12891-017-1502-8

**Published:** 2017-04-04

**Authors:** G. Mansell, K. P. Jordan, G. M. Peat, K. M. Dunn, D. Lasserson, T. Kuijpers, I. Swinkels-Meewisse, D. A. W. M. van der Windt

**Affiliations:** 1 Arthritis Research UK Primary Care Centre, Research Institute for Primary Care and Health Sciences, Keele University, Keele, Staffordshire ST5 5BG UK; 2 Nuffield Department of Medicine, University of Oxford and NIHR Oxford Biomedical Research Centre, John Radcliffe Hospital, Oxford, UK; 3 Department for Guideline Development, The Dutch College of General Practitioners, PO Box 3231, 3502 GE Utrecht, The Netherlands; 4 Centre for Physical Therapy and Rehabilitation, Geldrop, The Netherlands

**Keywords:** Prognosis, Primary care, Consultation, Musculoskeletal conditions, Low back pain, Shoulder pain

## Abstract

**Background:**

Research investigating prognosis in musculoskeletal pain conditions has only been moderately successful in predicting which patients are unlikely to recover. Clinical decision making could potentially be improved by combining information taken at baseline and re-consultation.

**Methods:**

Data from four prospective clinical cohorts of adults presenting to UK and Dutch primary care with low-back or shoulder pain was analysed, assessing long-term disability at 6 or 12 months and including baseline and 4–6 week assessments of pain. Baseline versus short-term assessments of pain, and previously validated multivariable prediction models versus repeat assessment, were compared to assess predictive performance of long-term disability outcome. A hypothetical clinical scenario was explored which made efficient use of both baseline and repeated assessment to identify patients likely to have a poor prognosis and decide on further treatment.

**Results:**

Short-term repeat assessment of pain was better than short-term change or baseline score at predicting long-term disability improvement across all cohorts. Short-term repeat assessment of pain was only slightly more predictive of long-term recovery (c-statistics 0.78, 95% CI 0.74 to 0.83 and 0.75, 95% CI 0.69 to 0.82) than a multivariable baseline prognostic model in the two cohorts presenting such a model (c-statistics 0.71, 95% CI 0.67 to 0.76 and 0.72, 95% CI 0.66 to 0.78). Combining optimal prediction at baseline using a multivariable prognostic model with short-term repeat assessment of pain in those with uncertain prognosis in a hypothetical clinical scenario resulted in reduction in the number of patients with an uncertain probability of recovery, thereby reducing the instances where patients may be inappropriately referred or reassured.

**Conclusions:**

Incorporating short-term repeat assessment of pain into prognostic models could potentially optimise the clinical usefulness of prognostic information.

**Electronic supplementary material:**

The online version of this article (doi:10.1186/s12891-017-1502-8) contains supplementary material, which is available to authorized users.

## Background

Musculoskeletal conditions account for 14% of all general practice (GP) consultations, with a quarter of the registered population consulting each year with a musculoskeletal problem [1]. Regional, non-inflammatory disorders - back, knee, neck and shoulder pain - are among the most common problems, together representing more than 40% of all consultations for musculoskeletal conditions [1]. Outcome varies considerably between individual patients [2–5] and remains difficult to predict accurately despite a considerable number of published studies deriving prognostic models [6]. This has led to uncertainty around how to manage patients who present to GPs with musculoskeletal pain. Such uncertainty may result in patients being referred unnecessarily, or being incorrectly reassured that their pain will subside when they might in fact benefit from early treatment.

Although the methods for prognostic model research have advanced over the past 10 years, little research has addressed issues related to the clinical usefulness and impact of prognostic information. A feature of most published studies in this field has been the reliance on information on prognostic factors assessed at one point in time (baseline), with prediction of future outcome based on this one assessment only. However, prognostic information will often include time-varying factors, and using information from a single assessment only is likely to result in suboptimal prognostic performance as patients tend to consult when their symptoms are at their worst, and short-term improvements following the consultation are likely [7]. These short-term improvements in pain episodes have been described not only in acute low back pain (e.g. [8]) but also in longer-term pain problems [9, 10]. A single assessment also seems out of touch with clinical practice where GPs incorporate information from previous visits to estimate prognosis and decide on treatment and referral. When a patient first consults with musculoskeletal pain it is not always certain whether they require immediate referral, can be reassured that their pain will subside, or require monitoring over time to see how the problem develops; further information gained from repeat consultations may help with such decisions. This implies that information collected at the time of the first consultation may not provide optimal prognostic information. Indeed, it has been previously shown that combining information on prognostic factors taken at baseline and short-term repeated assessment [11] provided better prediction of long-term outcome in low back pain patients than a baseline score. However, this does not address the issue of how to manage patients who re-attend primary care for musculoskeletal pain, or whether brief repeated assessments of symptoms provide more information about likely future outcomes compared to a more comprehensive baseline assessment. Research to address these questions might aid clinical decision making regarding the need for early referral and treatment in patients with a low probability of recovery, whether monitoring of symptoms might be helpful, or whether patients can be reassured regarding their prognosis. The aim of this study was therefore to explore whether the efficiency of clinical decision making can potentially be improved by combining information taken at baseline and re-consultation, as identifying optimal approaches to collecting prognostic data could lead to improved estimation of long-term outcomes, providing better information to support treatment decisions.

We hypothesised that incorporating information from repeated assessments of pain in prognostic models will significantly improve prognostic performance and reduce uncertainty in clinical decision making when estimating the likelihood of long-term outcomes in patients consulting with musculoskeletal pain.

### Specific objectives

1. To explore the prognostic performance of repeated (4–6 week) assessment or change in pain compared with baseline assessment only in the prediction of longer-term (6–12 month) outcome in painful musculoskeletal conditions, and evaluate the consistency of the findings across different samples;
2. To assess the prognostic performance of the incorporation of short-term repeated assessment of pain with data collected on multiple prognostic factors at baseline, and assess the unique contribution of short-term repeat assessment of pain, in the prediction of long-term outcome;
3. To test a strategy for accurately and efficiently classifying patients as being at low or high probability of recovery, based on prognostic information measured at a single point in time in combination with a short-term repeated assessment in those at intermediate risk of a poor prognosis at baseline.

## Methods

Potentially eligible studies were identified from two bibliographic databases held at Keele University that include the results of sensitive searches for prognosis studies in primary care. A systematic search of eligible studies was not conducted; the aim was instead to identify studies of different musculoskeletal conditions that met specific criteria, allowing us to test the study hypothesis. First authors or principal investigators of eligible studies were contacted to ask for their collaboration and request necessary data.

### Inclusion criteria

- Design: prospective cohort study;
- Population: patients consulting with spinal pain (back or neck), shoulder pain, or knee pain in primary care;
- Outcome measures: pain-specific disability (e.g. Roland-Morris Disability Questionnaire (RMDQ) [12] or Shoulder Disability Questionnaire (SDQ) [13]), patient-perceived recovery;
- Prognostic factor measurement: at least two assessments of pain: at baseline and short-term (within 4–6 weeks of baseline assessment);
- Outcome ascertainment: Longer-term follow-up (at least 6 months after baseline assessment).

### Statistical Analysis

Descriptive statistics were used to characterise each of the study samples, present long-term outcome scores for disability, and present short-term changes and repeat assessment scores in pain. Baseline and short-term pain scores were analysed on their original scale (e.g. visual analogue scale or 0–10 numeric rating scale [14]).

#### Objective 1: Comparison of repeat assessment with baseline value

The long-term recovery outcome for objective 1 was defined based on a validated cut-off point for minimal important change in disability for the RMDQ and SDQ (at least 30% improvement [15, 16]) allowing classification of participants into those “Improved” or “Not improved”.

For each study, pain measured at baseline was entered into separate logistic regression models with the dichotomised variable for disability improvement as the outcome measure, to investigate whether this baseline variable predicted improvement in disability at long-term follow-up. The same analysis was then repeated using short-term change (difference between baseline and shortest follow-up point from each study) and absolute short-term follow-up score (repeat assessment) of pain as predictors of disability improvement at long-term follow-up, in separate analyses. The strength of association (odds ratios (ORs) with 95% CI), model goodness-of-fit (Hosmer & Lemeshow and Nagelkerke pseudo r-square), and prognostic performance (AUC (c-statistic)) were compared for each model [17]. A c-statistic of 0.75 or above was considered to indicate good discrimination (i.e. a high level of sensitivity and specificity), and a value of 0.50 indicates a level of association no better than chance [17]. Differences in c-statistics were compared using the STATA roccomp command [18]. A *p* value of <0.05 indicates a statistically significant difference in the c-statistics.

#### Objective 2: Comparison of repeat assessment with multivariable baseline prognostic model

Only datasets that had already obtained a prediction model (van der Windt [19] and Kuijpers [20] – both shoulder pain populations) were used in this analysis. The outcome measure used was patient perceived recovery from shoulder symptoms (dichotomised: 1 = recovery) as used in the original prognostic model, rather than the disability improvement scores used in objective 1. Each of the prognostic models were re-run to check whether they produced the same results as the original study. Prognostic performance was then described and compared to that of incorporating a brief repeat assessment of pain in the same way as in Objective 1 (Hosmer & Lemeshow test, Nagelkerke pseudo R-square, ORs and AUC), using the STATA roccomp command to test for statistically significant differences in c-statistics between the models. We also examined the contribution of repeat assessment of pain when adding this to the original prognostic model using an omnibus test [21] to examine whether adding such short-term information significantly improved fit of the prediction model.

#### Objective 3: Scenario combining optimal baseline prediction with brief repeated assessment

**Fig. 1:**
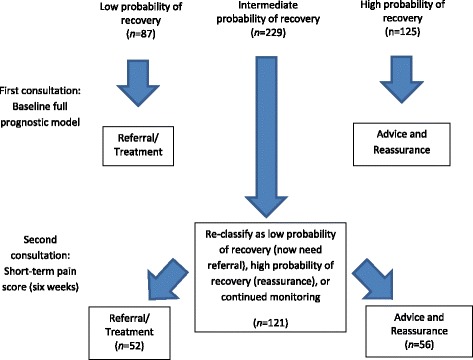
Clinical Scenario

The purpose of this final objective was to test a more efficient hypothetical clinical scenario (Fig. 1) using the Kuijpers [20] dataset, that does not require all patients to receive a repeat assessment. The scenario aims to discriminate between those with a high probability of recovery (i.e. likely to need advice and pain relief but no further monitoring required) and those with a low probability of recovery at six months (i.e. likely to require treatment or referral). Prognostic information collected at baseline in all patients was combined with a short-term repeat assessment of pain in those with an intermediate probability of recovery (uncertain prognosis). This scenario would result in only those who GPs were most uncertain about being asked to return for a repeat consultation, who would then be re-assessed and a better informed decision made based on the patient’s reporting of pain at that second consultation.

Patient perceived recovery at six months was used as the outcome measure (as in objective 2). Two stages of analyses were performed to assess prognostic performance of the combined strategy:

1. Discrimination at baseline between those at low, intermediate, and high probability of long-term recovery using cut-points based on predicted probabilities of recovery (scores of ≤0.33, 0.34 to 0.66, ≥0.67) from the optimal baseline prognostic model. Cut-offs for what constituted low, intermediate and high probability of recovery were chosen based on consensus among the clinicians and methodologists within the study team, given the absence of empirically derived cut-offs in this area.
2. Discrimination at short-term follow-up in those with intermediate probability of long-term recovery (*n* = 229) by reclassification as low, intermediate, and high probability of recovery at six months based on the results of the full baseline model plus a repeat assessment of pain six weeks after their baseline assessment. Cut-off points for predicted probabilities were the same as those used in step 1.

The results of these two stages were then compared to assess how many patients originally allocated to the intermediate probability (uncertain) group (stage 1) had moved to either the high or low probability group by short-term follow-up (stage 2), and how many of these were correctly or incorrectly classified in terms of their long-term outcomes.

## Results

### Descriptive Results

Four datasets were identified, requested and obtained [19, 20, 22, 23]. The design and results of these studies are summarised in Table 1. Two included patients with low back pain and two included patients with shoulder pain. No studies that included patients with knee pain were considered eligible for this study. The disability outcome measures used for these populations were the Roland-Morris Disability Questionnaire (RMDQ) [12] and Shoulder Disability Questionnaire (SDQ) [13] respectively. All studies recruited patients from primary care (general practice) clinics. One study [23] only included patients who had experienced pain for a month or less and two papers included patients with new pain episodes [19, 20]. The Dunn study [22] included a mix of patients with pain of short- and longer-term duration. All studies showed mean improvement in the outcome of disability, and 24% to 64% achieved 30% improvement in disability at the long-term follow-up point. The studies measured a number of different prognostic factors, including pain duration, fear-avoidance beliefs, [20, 22, 23] pain catastrophising [22] and pain coping [22]. Two studies [19, 20] had previously tested prognostic models within these datasets. Full details about the measures used in each study can be found in Additional file 1.

**Table 1:** Summary of Included Studies

Reference	Setting and study design	Sample	Follow-up	Outcome measures	Absolute scores (mean (SD)) on disability outcome	N(%) of participants meeting 30% improvement cut-off at long-term follow-up
Dunn & Croft 2006 [22]	Primary care; Prospective cohort	430 patients with LBP (mean age 44.7 years; 47% male)	Four weeks and 12 months	Disability (RMDQ) – score of between 0–24 with a higher score indicating higher disability	Baseline: 9.02 (6.63) 4 weeks: 7.48 (6.48) 12 months: 6.61 (6.74)	228 (24) at 12 months
Kuijpers et al. 2006 [20]	Primary care; prospective cohort	512 patients with a new episode of shoulder pain (mean age 51.49 years; 49.7% male)	Six weeks and six months	Recovery: Two categories (1: 0 = Not recovered and 1 = Recovered; 2: Global perceived recovery based on a 7-point Likert scale (6 = very much improved to 0 = very much deteriorated)) Disability (SDQ – score of between 0–100 – higher score indicating higher disability)	Baseline: 59.89 (24.21) 6 weeks: 40.32 (30.28) 6 months: 27.30 (31.26)	354 (60) at six months
Swinkels-Meewisse et al. 2006 [23]	Primary care; Prospective inception cohort	300 patients with acute LBP (less than four weeks duration) (mean age 43.01 years; 58.2% male)	Six weeks and six months	Disability (RMDQ, see Dunn)	Baseline: 13.15 (5.02) 6 weeks: 6.15 (5.45) 6 months: 4.42 (5.17)	303 (55) at six months
van der Windt et al. 1996 [19]	Primary care; Prospective cohort	300 patients with a new episode of shoulder pain (mean age 49.52 years; 44.4% male)	Four weeks and 12 months	Recovery: Two categories (1: 0 = Persisting symptoms and 1 = Full recovery/much improved; 2: Global perceived recovery based on a 5-point Likert scale (5 = Much improved to 0 = Much deteriorated)) Disability (SDQ, see Kuijpers)	Baseline: 66.6 (23.4) 4 weeks: 47.1 (31.3) 12 months: 29.0 (30.3)	214 (64) at 12 months

#### Objective 1

This objective compared the performance of the different models in predicting long-term disability outcome: (1) baseline pain scores only, (2) short-term pain score (repeat assessment at short-term follow-up), and (3) short-term change in pain (change since baseline). The Hosmer & Lemeshow tests indicated good fit for nearly all analyses (Table 2). The Nagelkerke R^2^ values were higher for the repeat assessment of pain models. The c-statistics (AUC) also suggest that a repeat assessment of pain at short-term follow-up best predicts the outcome of disability improvement, followed by the short-term change models. For three of the datasets (Dunn, [22] Kuijpers, [20] and Swinkels-Meewisse [23]), statistically significant differences (*p* < 0.05) were found between the c-statistics for the models using baseline pain and repeat assessment score. A statistically significant difference in c-statistics was found between repeat assessment score and change in pain in two of the datasets (Swinkels-Meewisse [23] and Van der Windt [19]). Overall, repeat assessment of pain appeared to best predict disability outcome.

**Table 2:** Strength of association (odds ratios, 95% CI), goodness-of-fit statistics and discrimination (c-statistic) for prediction models using baseline pain only, short-term change, or repeat score at repeat assessment (long-term disability improvement as outcome)

Study Author	Prediction model	OR (95% CI)	Goodness-of-fit statistics		c-statistic (95% CI)	Comparison of c-statistics (*p*-*value*)
			Hosmer & Lemeshow test	Nagelkerke pseudo R square		
Dunn & Croft [22] LBP, RMDQ outcome	Baseline Pain Score (0–10) (*n* = 430)	0.94 (0.88 to 1.01)	*X* ^2^_(7)_ = 8.18, *p* = 0.32	0.01	0.55 (0.49 to 0.60)	Baseline vs. 4w pain, p < 0.001* Baseline vs. Change, p = 0.24 Change vs. 4w pain, p = 0.06
	4w** Change in Pain (*n* = 332)	1.21 (1.10 to 1.33)*	*X* ^2^_(6)_ = 4.85, *p* = 0.56	0.07	0.62 (0.56 to 0.68)	
	4w Pain Score (*n* = 334)	0.78 (0.72 to 0.85)*	*X* ^2^_(7)_ = 13.48, *p* = 0.06	0.13	0.68 (0.63 to 0.74)	
Kuijpers et al. [20] Shoulder pain, SDQ outcome	Baseline Pain Score (0–10) (*n* = 586)	0.92 (0.85 to 0.98)*	*X* ^2^_(6)_ = 6.10, *p* = 0.41	0.01	0.56 (0.51 to 0.61)	Baseline vs. 6w pain, p <0.001* Baseline vs. Change, p = 0.30 Change vs. 6w pain, p = 0.02*
	6w Change in Pain (*n* = 477)	1.17 (1.09 to 1.26)*	*X* ^2^_(7)_ = 5.09, *p* = 0.65	0.05	0.61 (0.56 to 0.67)	
	6w Pain Score (*n* = 478)	0.78 (0.72 to 0.85)*	*X* ^2^_(6)_ = 3.68, *p* = 0.72	0.11	0.67 (0.62 to 0.72)	
Swinkels-Meewisse et al. [23] LBP, RMDQ outcome	Baseline Pain Score (0–10) (*n* = 300)	0.99 (0.98 to 1.01)	*X* ^2^_(8)_ = 6.99, *p* = 0.54	0.01	0.56 (0.47 to 0.64)	Baseline vs. 6w pain, p <0.001* Baseline vs. Change, p = 0.03* Change vs. 6w pain, p = 0.09
	6w Change in Pain (*n* = 279)	1.03 (1.02 to 1.04)*	*X* ^2^_(8)_ = 11.20, *p* = 0.19	0.13	0.71 (0.63 to 0.79)	
	6w Pain Score (*n* = 281)	0.97 (0.95 to 0.98)*	*X* ^2^_(8)_ = 6.25, *p* = 0.62	0.20	0.77 (0.70 to 0.84)	
van der Windt et al. [19] Shoulder pain, SDQ outcome	Baseline Pain Score (0–10) (*n* = 293)	0.83 (0.74 to 0.94)*	*X* ^2^_(5)_ = 5.24, *p* = 0.39	0.05	0.62 (0.55 to 0.69)	Baseline vs. 4w pain, p = 0.44 Baseline vs. Change, p = 0.22 Change vs. 4w pain, p <0.001*
	4w Change in Pain (*n* = 280)	1.07 (0.97 to 1.16)	*X* ^2^_(7)_ = 7.04, *p* = 0.43	0.01	0.55 (0.48 to 0.63)	
	4w Pain Score (*n* = 287)	0.83 (0.75 to 0.91)*	*X* ^2^_(6)_ = 3.50, *p* = 0.74	0.08	0.65 (0.58 to 0.72)	

*p < 0.05; **change calculated as baseline minus 4w score; therefore an OR > 1 indicates a larger probability of long-term improvement and an OR < 1 indicates a smaller probability of improvement

#### Objective 2

In the two datasets used for objective 2 (Van der Windt [19] and Kuijpers [20]), the number of people reporting recovery was 53% (*n* = 232) in the Kuijpers [20] dataset and 66% (*n* = 218) in the Van der Windt [19] dataset. When the models (full prediction model at baseline, and full prediction model incorporating repeat assessment of pain) for each dataset were compared, the c-statistics indicated that the addition of repeat assessment of pain was slightly more predictive compared to the full baseline models only, with the difference in predictive performance statistically significant (*p* < 0.05) for the Kuijpers dataset (c-statistic 0.78, 95% CI 0.74 to 0.83 versus 0.71, 95% CI 0.67 to 0.76), but not for the Van der Windt dataset (c-statistic 0.75, 95% CI 0.69 to 0.82 versus 0.72, 95% CI 0.66 to 0.78, see Table 3). The omnibus tests for both datasets indicate that the addition of repeat assessment of pain (Kuijpers *X* ^2^_(6)_ = 109.23, *p* < 0.05; van der Windt *X* ^2^_(4)_ = 35.00, *p* < 0.05) to the full baseline prognostic model significantly improve the fit of the model.

**Table 3:** Strength of association (odds ratios, 95% CI), goodness-of-fit statistics, and discrimination for prediction models using a baseline multivariable prognostic model versus baseline pain only, short-term change or repeat score at repeat assessment – long-term perceived recovery as outcome

Study author	Prediction model	OR (95% CI)	Goodness-of-fit statistics		c-statistic (95% CI)	Comparison of c-statistics (*p-value*)
			Hosmer & Lemeshow test	Nagelkerke pseudo R square		
Kuijpers	*Full model (n = 441)* Baseline pain (0–10) Duration of complaint: - less than 5 weeks - 6–11 weeks - more than 3 m Concomitant LBP (yes/no) Shoulder pain score at physical examination (0–14) Gradual onset (yes/no)	ᅟ 0.89 (0.82 to 0.98)* ᅟ 1 0.68 (0.40 to 1.18) 0.36 (0.22 to 0.60)* 0.52 (0.32 to 0.84)* 0.96 (0.91 to 1.01) ᅟ 0.60 (0.39 to 0.95)*	ᅟ *X* ^2^_(8)_ = 10.45, *p* = 0.24	ᅟ 0.19	ᅟ 0.71 (0.67 to 0.76)	
	Full model plus 6w Pain Score (*n* = 441)	0.72 (0.65 to 0.80)*	*X* ^2^_(8)_ = 10.89, *p* = 0.21	0.29	0.78 (0.74 to 0.83)	Full model vs. full model plus 6w pain score, *p* <0.001*
Van der Windt	*Full model (n = 282)* Baseline Pain (0–10) Co-existing neck pain (yes/no) Preceding trauma (yes/no) Diagnosis Acute Bursitis (yes/no)	ᅟ 0.80 (0.69 to 0.91)* 0.47 (0.27 to 0.82)* 7.44 (1.69 to 32.85)* 2.32 (1.03 to 5.20)*	ᅟ *X* ^2^_(8)_ = 9.05, *p* = 0.34	ᅟ 0.17	ᅟ 0.72 (0.66 to 0.78)	
	Full model plus 4w Pain Score (*n* = 270)	0.85 (0.76 to 0.95)*	*X* ^*2*^_(8)_ = 15.07, *p* = 0.06	0.22	0.75 (0.69 to 0.82)	Full model vs. full model plus 4w pain score, *p* = 0.10

*p < 0.05

#### Objective 3

The purpose of this final objective was to explore the hypothetical clinical scenario, aiming to combine optimal baseline data with follow-up assessment data in a subset of participants with uncertain prognosis, which would make efficient use of the repeated assessment. The full prediction model was found to provide good prognostic performance at baseline in the Kuijpers [20] dataset (Table 3). Table 4 shows the estimated frequencies of the low, intermediate and high probability of recovery groups in the two stages, and their observed outcome frequencies. Figure 1 presents the numbers in the context of the scenario for clarity.

**Table 4:** Frequencies of low, intermediate and high probability of recovery and observed long-term recovery when classifying shoulder pain patients at baseline based on the full prediction model

Hypothetical scenario	Classification	Frequency *n*(%)	Perceived recovery at six months	
			Not recovered *N* = 209 *n*(%)	Recovered *N* = 232 *n*(%)
Stage 1 Full prediction model at baseline (*n* = 441)	1 – Low probability of recovery	87 (19.7)	64 (73.6)	23 (26.4)
	2 – Intermediate probability of recovery	229 (51.9)	116 (50.7)	113 (49.3)
	3 – High probability of recovery	125 (28.3)	29 (23.2)	96 (76.8)
Stage 1&2 Full model plus re-assessment of pain at six weeks – Intermediate group only (*n* = 229)	1 – Low probability of recovery	52 (22.7)	40 (76.9)	12 (23.1)
	2 – Intermediate probability of recovery	121 (52.8)	67 (55.4)	54 (44.6)
	3 – High probability of recovery	56 (24.5)	9 (16.1)	47 (83.9)
Model Stage 1&2 Full model plus re-assessment of pain at six weeks (*n* = 441)	1 – Low probability of recovery	87 + 52 = 139 (31.5)	64 + 40 = 104 (74.8)	23 + 12 = 35 (25.2)
	2 – Intermediate probability of recovery	121(27.4)	67 (55.4)	54 (44.6)
	3 – High probability of recovery	125 + 56 = 181 (41.0)	29 + 9 = 38 (21.0)	96 + 47 = 143 (79.0)

In stage 1, the full model at baseline presentation leaves 229 (52%) participants in the uncertain (intermediate) category. Repeat assessment of pain at six weeks in this intermediate group reduces this number to 121 (27.4%), reclassifying an equal number to low or high probability of recovery (*n* = 52 and 56 respectively) in stage 2. These results suggest that the final combined model is slightly better at identifying participants at high and low probability of recovery (79% and 75% of patients in these two groups correctly classified respectively) than the stage 1 model. The combined model misclassifies 18% of those who did not recover (38/209 would have been incorrectly reassured) and 15% of those who did recover (35/232 would have been unnecessarily referred).

## Discussion

### Summary of findings

The aim of this study was to investigate whether the use of data from repeated assessments enhanced model fit and prognostic performance of primary care prognostic models, in order to better predict long-term outcomes and reduce uncertainty in clinical decision-making. Such information can support decision making regarding the need for monitoring symptoms, or immediate referral or treatment. The results show that short-term repeat assessment of pain had only slightly better prognostic performance compared to short-term change or baseline only scores in predicting long-term disability improvement. Furthermore, a full baseline prediction model incorporating multiple prognostic factors does not necessarily add prognostic value to a repeat short-term assessment of pain. Using a hypothetical clinical scenario, combining information from a full prediction model assessed at baseline with a brief repeat assessment of pain in only those with uncertain prognosis at initial assessment may provide an efficient and effective strategy towards reducing uncertainty and improving discrimination between those at high or low probability of long-term recovery from a painful shoulder condition.

### Comparison with existing literature

The results of this study do partially confirm those of two similar studies [8, 11] which also examined the added prognostic value of short-term monitoring on longer-term outcomes. Dunn & Croft [11] used the same dataset as one of those used in the present study, and found that repeat assessment of other prognostic factors (e.g. fear-avoidance beliefs) led to an improvement in predictions longer-term. The similarity between this previous study and the findings from the other datasets included here strengthens the argument that repeat assessment can provide additional prognostic information across different outcome measures. Wand et al. [8] found that a ‘subacute profile’ (predictors measured at six weeks) was the best at predicting long-term disability compared to an ‘acute profile’ (baseline scores only) and compared to change between baseline and six-week assessment. Our study extended the findings of both of these studies by testing a possible clinical scenario in which a more efficient strategy (only re-assessing those with uncertain prognosis rather than the entire sample) could be applied.

The highest c-statistics obtained in objective 2 are not as high as those reported for other prognostic models [2–5]. For example, the STarT Back tool which can be used to stratify patients with low back pain [24] has demonstrated better prognostic performance (e.g. [25, 26]). Variability in performance can be explained by differences in the outcome measure used in each study (function versus recovery), prognostic factors included in the model, the measures used to assess prognostic factors, or differences in the study population. However, these other studies could not be used as they did not include a short-term assessment point which we required in the present analysis. The aim of our analysis was not to find the “best” model, but rather to find the most appropriate studies to test our hypotheses and clinical scenario for incorporating repeat assessment. Further research is required to confirm whether this improvement holds for other baseline prognostic models, regardless of prognostic performance.

### Strengths and Limitations

This study included four relatively large (*n* at least 512) datasets covering two common musculoskeletal complaints. The studies included different measures of disability and used slightly different measurement time points, but the results overall were very similar, which gives strength to the study findings [27]. The number of cases included in each of the analyses did differ, due to dropout over time in each of the studies. Imputation was considered, but a sensitivity analysis using complete case data did not result in any significant differences in results. The sample sizes available for each of the datasets included in this study did meet guidance around numbers of cases required for building prognostic models of an event to predictor ratio of at least 10 (e.g. [28]), although we realise that such guidance may not be entirely suitable for the specific analysis performed in this study.

The inclusion of different musculoskeletal pain regions could be considered problematic as shoulder pain problems may have a different rate of recovery, and different prognostic factors compared to back pain problems. However, recent cohort studies and systematic reviews have highlighted similarities in symptom trajectories and identified generic prognostic factors across different regional musculoskeletal pain problems (e.g. [6, 29, 30]). This is supported by our findings from objective 1, confirming the prognostic value of short-term changes in pain for predicting long-term disability outcomes in both back and shoulder pain.

The included studies were selected according to specific criteria to test the present study hypothesis, but were not systematically searched for. This means that we may have missed additional studies that could have been included in our analysis. However, a search of the wider literature found that very few prognosis studies set in primary care include a short-term assessment point, which was a requirement for our analysis. We would however welcome further analysis of the prognostic value of repeat pain assessment in other settings or populations.

Not every dataset could be used for analysis of each objective. While both the Van der Windt and Kuijpers datasets included a prognostic model, differences between these models in terms of the proportion of participants recovered (outcome event rate) would require different classifications for predicted probabilities in order to address objective 3, making them difficult to compare. Only the Kuijpers model was therefore used for this analysis to provide an example for how such a strategy may be used in clinical practice and how researchers can investigate prognosis in the future. To strengthen these findings, replication in other datasets and other musculoskeletal conditions is needed, perhaps especially because prognostic performance of the models presented here could be considered as moderate at best.

The datasets examined here only contain patients who consulted for musculoskeletal pain, and provided data at both baseline and follow-up, and therefore may not be representative of people who have musculoskeletal pain but choose not to consult, or would not re-consult when invited for a repeat assessment. The datasets included in this study did not all take baseline assessments at the point of consultation. As most patients are likely to consult when their pain is at its worst, and may therefore experience a natural reduction in pain shortly after consulting, the scores obtained in these studies may give a different interpretation and prognostic value than if scores were obtained during the consultation. Studies investigating prognostic factors at the point of care found that pain reduced shortly following consultation, [31] suggesting that the point at which measures are taken is important and could affect the accuracy of any prediction models derived [31].

The clinical scenario presented here does not offer optimum prediction. However, this version of the scenario (with three equal risk categories), while realistic for this example, was meant as an illustration of this approach; it could be that different cut-off points for low, intermediate and high probability would result in fewer people being asked to re-consult. The choice of cut-off point should therefore depend on clinically relevant thresholds for treatment and referral. The number of patients in the intermediate group at baseline who needed to return for repeat assessment was still large in our scenario, highlighting the large amount of uncertainty in the prognosis of musculoskeletal pain conditions. Similar proportions for intermediate groups were used in the STarT Back study, [24] which included active management of this group rather than a ‘watch and wait’ scenario, although in the STarT Back study these proportions were a reflection of the patients included rather than pre-specified proportions as in the present study. The scenario presented here builds on this by ensuring that the low- and high-probability groups are optimally identified, either at first consultation or within a few weeks of that first consultation. It could be that a stronger prognostic model with better prognostic performance is needed in order to more clearly differentiate between patients who do or do not require referral.

The hypothetical scenario itself, while aiming to reduce the number of inappropriate clinical decisions, may also lead to an increase in consultations with an impact on GP time and costs. It could be that the single question about pain could be asked via a phone call, SMS text message/smart phone application 4–6 weeks after the initial consultation, with only those who still report pain at that point being invited back to see their GP.

## Conclusions

This study investigated a clinical scenario (Fig. 1) that could be used to help reduce the uncertainty of clinical decision-making in GP consultations with a patient with musculoskeletal pain. This scenario shows a decrease in unnecessary referrals by proposing a brief (single question), short-term repeat assessment in those where prognosis remains uncertain at the first consultation. It should also be acknowledged that such a strategy has costs too, such as potential delay in treatment, cost of repeat assessment, and the need to have a system for combining baseline and repeat assessment measures, which might make such a strategy unsuitable in some circumstances. However, the importance of this study lies in its shift towards reducing uncertainty and enabling clinicians to more confidently refer or reassure people at an early stage using minimal information.

Prognostic models would potentially benefit from the inclusion of short-term assessments of key prognostic factors, in addition to baseline assessments. Further research should investigate to what extent the proposed strategy improves management compared to more extensive data collection at a single point in time (baseline), across different health conditions, in order to further test the added value of monitoring for estimating prognosis and optimising clinical decision making.

## Additional file

Additional file 1: Changes over time (Means, SDs and percentages) and descriptive data for outcome and predictor variables. (DOCX 21 kb)
