# Supplementary material for: Brief pain re-assessment provided more accurate prognosis than baseline information for low-back or shoulder pain
Source: BMC Musculoskelet Disord. 2017 Apr 4;18:139. doi: 10.1186/s12891-017-1502-8 (PMC5379746; doi:10.1186/s12891-017-1502-8)
Supplement: Additional file 1: — Changes over time (Means, SDs and percentages) and descriptive data for outcome and predictor variables. (DOCX 21 kb) [file 12891_2017_1502_MOESM1_ESM.docx]

Additional file 1. Changes over time (Means, SDs and percentages) and descriptive data for outcome and predictor variables – Dunn Data

| **Measure** | | | **Baseline *n*=935** | **Short-term Follow-up (4 weeks), *n*=484** | **Long-term Follow-up (12 months), *n*=466** |
| --- | --- | --- | --- | --- | --- |
| Age | | | 44.7 | - | - |
| Sex | | | 47% male | - | - |
| Pain duration (last pain-free month) | <3 months | | 199 (13.5%) | - | - |
|  | 3-6 months | | 120 (8.2%) | - | - |
|  | 7-12 months | | 101 (6.9%) | - | - |
|  | 1-2 years | | 146 (9.9%) | - | - |
|  | 3-5 years | | 129 (8.8%) | - | - |
|  | 6-10 years | | 98 (6.7%) | - | - |
|  | >10 years | | 124 (8.4%) | - | - |
| Disability (RMDQ) | | | 9.02 (SD 6.63) | 7.48 (SD 6.48) | 6.61 (SD6.74) |
| Pain Intensity (NRS) | Pain right now | | 4.55 (SD 2.93) | 3.68 (SD 3.09) | 3.34 (SD 3.05) |
|  | Average pain | | 5.36 (SD 2.87) | 4.05 (SD 2.73) | 3.68 (SD 2.95) |
| Anxiety | | | 8.55 (SD 4.86) | *** | *** |
| Depression | | | 7.11 (SD 4.63) | *** | *** |
| Fear-avoidance | | Y | 204 (13.9%) | 106 (7.2%) | *** |
|  |  | N | 731 (49.7%) | 378 (25.7%) | *** |
| Catastrophising | | Y | 260 (17.7%) | 104 (7.1%) | *** |
|  |  | N | 675 (45.9%) | 380 (25.8%) | *** |
| Pain coping | | Y | 592 (40.2%) | 280 (19%) | *** |
|  |  | N | 343 (23.3%) | 204 (13.9%) | *** |

Change over time (Means, SDs and percentages) for Outcome and Predictor Variables – Kuijpers Data

| **Measure** | | **Baseline**  ***n*=587** | **Short-term Follow-up (6 weeks)**  ***n*=486** | **Long-term Follow-up (6 months)**  ***n*=533** |
| --- | --- | --- | --- | --- |
| Age | | 51.49 (SD 14.02) | - | - |
| Sex | | 49.7% male | - | - |
| Pain duration | 0-5w | 205 (34.9%) | - | - |
|  | 6-11w | 139 (23.7%) | - | - |
|  | >3m | 242 (41.2%) | - | - |
| Pain intensity | | 4.79 (SD 2.32) | 3.12 (SD 2.51) | 2.14 (SD 2.56) |
| Disability (SDQ) | | 59.89 (SD 24.21) | 40.32 (SD 30.28) | 27.30 (SD 31.26) |
| Coping (PCCL) | | 2.98 (SD 0.98) | 3.11 (SD 1.09) | - |
| Anxiety (PCCL) | | 0.33 (SD 1.24) | - | - |
| Depression (PCCL) | | 0.23 (SD 1.28) | - | - |
| Somatisation (PCCL) | | 3.34 (SD 4.12) | - | - |
| Internal pain control | | 3.33 (SD 0.92) | - | - |
| External pain control | | 3.21 (SD 0.88) | - | - |
| Catastrophising (PCCL) | | 2.23 (SD 0.83) | 1.96 (SD 0.83) | - |
| Fear-avoidance beliefs (FABQ) | | 14.13 (SD 5.58) | 9.17 (SD 5.37) | - |

Change over time (means, SDs and percentages) and descriptive data for outcome and predictor variables – Swinkels-Meewisse Data

| **Measure** | | **Baseline**  ***n*=615** | **Short-term Follow-up (6 weeks)**  ***n*=519** | **Long-term Follow-up (6 months)**  ***n*=368** |
| --- | --- | --- | --- | --- |
| Age | | 43.01 (SD 11.06) | - | - |
| Sex | | 58.2% male | - | - |
| Pain duration | <7 days | 216 (35.1%) | - | - |
|  | 7-14 days | 163 (26.5%) | - | - |
|  | 14-21 days | 113 (18.4%) | - | - |
|  | 21-28 days | 122 (19.8%) | - | - |
| Fear avoidance (TSK) | | 36.62 (SD 7.55) | 34.20 (SD 7.33) | 34.13 (SD 8.07) |
| Disability (RMDQ) | | 13.15 (SD 5.02) | 6.15 (SD 5.45) | 4.42 (SD 5.17) |
| Pain intensity (VAS) | | 49.97 (SD 24.55) | 20.40 (SD 23.18) | 16.58 (SD 23.49) |
| Fear-avoidance beliefs: Physical activity subscale | | 13.71 (SD 5.74) | 11.49 (SD 6.07) | 10.60 (SD 6.30) |
| Fear-avoidance beliefs: Work subscale | | 14.82 (SD 10.41) | 12.25 (SD 10.13) | 11.19 (SD 10.76) |

Change over time (means, SDs and percentages) for Outcome and Predictor Variables – van der Windt Data

| **Measure** | | **Baseline**  ***n*=333** | **Short-term Follow-up (4 weeks), *n*= 321** | **Long-term Follow-up (12 months), *n*=302** |
| --- | --- | --- | --- | --- |
| Age | | 49.52 (SD 14.28) | - | - |
| Sex | | 55.6% female | - | - |
| Pain Duration | <1w | 47 (14.1%) | - | - |
|  | 1w-1m | 118 (35.4%) | - | - |
|  | 1m-6m | 113 (33.9%) | - | - |
|  | >6m | 50 (15.0%) | - | - |
| Disability | | 66.6 (SD 23.4) | 47.1 (SD 31.3) | 29.0 (SD 30.3) |
| Pain Intensity (NRS) | | 7.17 (SD 2.43) | 4.35 (SD 2.82) | 2.72 (SD 2.65) |
